# Public health round-up

**DOI:** 10.2471/BLT.23.010223

**Published:** 2023-02-01

**Child nutrition crisis**

A girl drinks water at a stabilization centre in Sudan where she was admitted with severe acute malnutrition in August 2022. Sudan is one of 15 countries identified in a recent interagency call for action to address an ongoing nutrition crisis that threatens the health of more than 30 million children.

**Figure:**
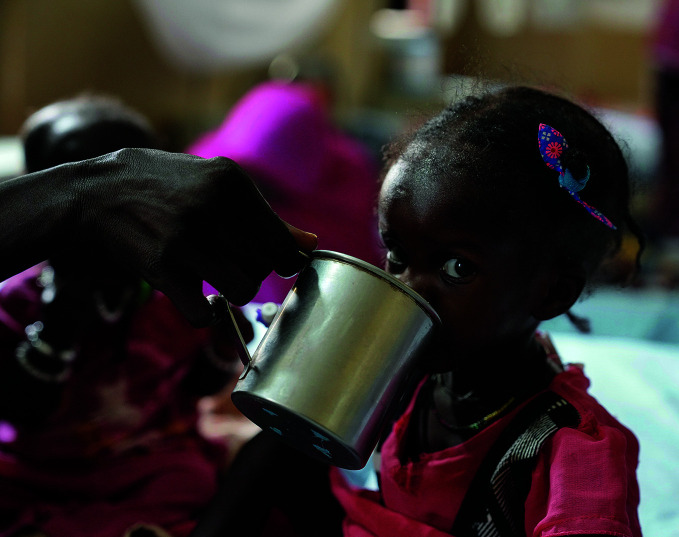
UNICEF/Mustafa Abdalrasol

## Call for action on child nutrition

United Nations (UN) agencies called for urgent action to protect the most vulnerable children in the 15 countries hardest hit by an ongoing nutrition crisis that is being driven by conflict, climate shocks, the coronavirus disease 2019 (COVID-19) pandemic, and rising food prices.

According to a statement released on 12 January, more than 30 million children in the 15 worst-affected countries (Afghanistan, Burkina Faso, Chad, Democratic Republic of the Congo, Ethiopia, Haiti, Kenya, Madagascar, Mali, Niger, Nigeria, Somalia, South Sudan, Sudan and Yemen) suffer from wasting – or acute malnutrition; 8 million of those children are assessed to be severely wasted.

The UN agencies called for accelerated implementation of the Global Action Plan on Child Wasting, identifying five priority actions.

“The global food crisis is also a health crisis, and a vicious cycle: malnutrition leads to disease, and disease leads to malnutrition,” said World Health Organization (WHO) Director-General Tedros Adhanom Ghebreyesus. “Urgent support is needed now in the hardest hit countries to protect children’s lives and health, including ensuring critical access to healthy foods and nutrition services, especially for women and children.”

https://bit.ly/3QvYy7z

## Uganda declares end of Ebola outbreak

Uganda declared the end of the Ebola disease outbreak caused by the Sudan ebolavirus strain. The announcement was made on 11 January, less than four months after the first case was confirmed in the Mubende district on 20 September 2022.

Jane Ruth Aceng Ocero, Uganda’s Minister of Health, credited the rapid ramping up of key control measures with bringing the outbreak to a swift close, while also underlining the importance of effective community action.

WHO and partners supported Ugandan health authorities from the outset, deploying experts, providing training in contact tracing, testing and patient care, as well as building isolation and treatment centres and providing laboratory testing kits.

WHO also helped to protect frontline health workers by supplying personal protective equipment, nearly US$ 6.5 million to support response activities, and an additional US$ 3 million to support outbreak readiness in six neighbouring countries.

https://bit.ly/3CC49U8

## Maintaining the COVID-19 response

Hans Henri Kluge, WHO Regional Director for Europe, called for countries across Europe and central Asia to avoid complacency in responding to the ongoing COVID-19 pandemic.

In a statement released on 10 January, Kluge emphasized the importance of re-investing in and recommitting to enhanced virologic and genomic surveillance – including wastewater surveillance, where feasible – and investing in and safeguarding health professionals, whose precarious situation in many places threatens to undermine the effective provision of health services.

https://bit.ly/3CLsxm7

## COVID-19 in China

In related news, WHO sought clarification with regard to the COVID-19 epidemic in mainland China, meeting with high-level officials from China on 30 December 2022. Chinese scientists presented data on viral sequencing at a meeting of the severe acute respiratory syndrome coronavirus 2 (SARS-CoV-2) Technical Advisory Group on Virus Evolution on 3 January.

On 14 January, WHO Director-General Tedros Adhanom Ghebreyesus spoke about the COVID-19 situation in the country with Minister Ma Xiaowei, director of China’s National Health Commission, and Chinese officials released information on a range of indicators, including in-hospital deaths related to COVID-19 infections, the number of patients admitted to clinics and hospitals, and the number requiring emergency treatment and critical care.

WHO requested a more detailed breakdown of data by province over time and called for such information to be made public on a continuing basis.

http://bit.ly/3iHGniI

https://bit.ly/3XlIpnd

## WHO updates COVID-19 guidance

WHO issued updated guidelines on mask wearing, COVID-19 treatments and clinical management. Issued on 13 January, the guidelines recommend mask wearing following a recent exposure to COVID-19, when someone has or suspects they have COVID-19, when someone is at high-risk of severe COVID-19, and for anyone in a crowded, enclosed or poorly ventilated space.

The guidelines take note of evidence indicating that asymptomatic people are much less likely to transmit the virus than those with symptoms and update recommendations regarding isolation.

With regard to COVID-19 treatments, the guidelines maintain a strong recommendation for the use of nirmatrelvir-ritonavir which was first recommended by WHO in April 2022, and the first generic producer of which was prequalified by WHO in December 2022.

http://bit.ly/3QIt9yU

## UN calls for inclusion of women

The principals of the Inter-Agency Standing Committee (the highest-level humanitarian coordination forum of the UN system) on Afghanistan, called for Afghanistan’s de facto authorities to reverse the directive to ban women from working in humanitarian nongovernmental organizations. The committee also called for the reversal of other directives issued by the authorities banning women from schools, universities and public life.

In a statement issued 28 December, the committee pointed out that female staff are key to delivering humanitarian interventions in Afghanistan in roles ranging from organizational directors and doctors to nurses and community health workers. Crucially, they can access populations that their male colleagues cannot reach and are thus absolutely indispensable.

https://bit.ly/3vSYAwv

## Unreliable electricity for health

Close to 1 billion people in low- and lower-middle-income countries are served by health-care facilities with unreliable electricity supply or no electricity at all. This is according to a report published by WHO, the World Bank, the International Renewable Energy Agency and Sustainable Energy for All on 14 January.

*Energizing health: accelerating electricity access in health-care facilities* presents the latest data on electrification of health-care facilities in low- and middle-income countries. It also projects investments required to achieve adequate and reliable electrification in health care and identifies priority actions for governments and development partners.

http://bit.ly/3w4cxrK

## New treatment options for drug-resistant tuberculosis

WHO released updated consolidated guidelines on the treatment of drug-resistant tuberculosis (DR-TB) featuring improved treatment options for people with multidrug-resistant or rifampicin-resistant tuberculosis (MDR/RR-TB).

Released 15 December, the guidelines include a new recommendation on the use of a novel all-oral 6-month regimen which offers better outcomes and significantly shortens the duration of treatment, thus improving the quality of life for people with MDR/RR-TB.

To facilitate and promote implementation of the new regimen, WHO will set up a regular online discussion platform that will include high MDR/RR-TB burden countries, civil society, technical partners and the donor community.

https://bit.ly/3X4rNAz

**Cover photo**

Firefighters tackle a wildfire in Pontevedra, Spain in August 2022 – the fire went on to burn at least 400 hectares of land along the border between Spain and Portugal.

**Figure:**
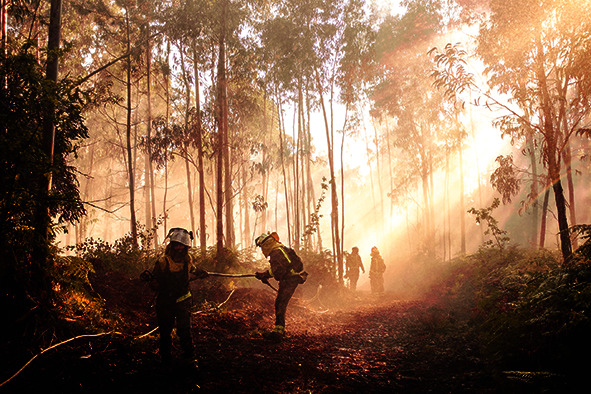
WHO/NOOR/Sebastian Liste

## New child and youth mortality data

An estimated 5 million children died before their fifth birthday in 2021 according to the latest estimates released by the UN Inter-Agency Group for Child Mortality Estimation. The deaths represent an under-five mortality rate (U5MR) of 38 deaths per 1000 live births.

Published 11 January, *Levels & trends in child mortality* reveals that U5MR has been falling since 1990, but gains have reduced significantly since 2010, and 54 countries will fall short of meeting the sustainable development goals under-five mortality target.

In a separate report, also released on 11 January, the group found that 1.9 million babies were stillborn during the same period, many of whom could have been saved.

The two reports are the first of a series of data sets to be released in 2023, with UN maternal mortality figures to be published later this year.

https://bit.ly/3VXnJB3

**Upcoming events**

23–24 February. Fifth Global Ministerial Summit on Patient Safety 2023, Montreux, Switzerland. https://bit.ly/3knSxOk

3 March. World hearing day, highlighting the importance of integrating ear and hearing care within primary care. http://bit.ly/3Wf2VF2

3–5 April. Fifth Global Forum on Human Resources for Health, Geneva, Switzerland. http://bit.ly/3kjKQsm

